# Enhancing Integrated Pest Management in GM Cotton Systems Using Host Plant Resistance

**DOI:** 10.3389/fpls.2016.00500

**Published:** 2016-04-22

**Authors:** Carlos Trapero, Iain W. Wilson, Warwick N. Stiller, Lewis J. Wilson

**Affiliations:** ^1^CSIRO Agriculture, Narrabri, NSWAustralia; ^2^CSIRO Agriculture, Canberra, ACTAustralia

**Keywords:** *Gossypium*, genetic resistance, plant breeding, resistance traits, plant defense mechanisms, arthropod control

## Abstract

Cotton has lost many ancestral defensive traits against key invertebrate pests. This is suggested by the levels of resistance to some pests found in wild cotton genotypes as well as in cultivated landraces and is a result of domestication and a long history of targeted breeding for yield and fiber quality, along with the capacity to control pests with pesticides. Genetic modification (GM) allowed integration of toxins from a bacteria into cotton to control key Lepidopteran pests. Since the mid-1990s, use of GM cotton cultivars has greatly reduced the amount of pesticides used in many cotton systems. However, pests not controlled by the GM traits have usually emerged as problems, especially the sucking bug complex. Control of this complex with pesticides often causes a reduction in beneficial invertebrate populations, allowing other secondary pests to increase rapidly and require control. Control of both sucking bug complex and secondary pests is problematic due to the cost of pesticides and/or high risk of selecting for pesticide resistance. Deployment of host plant resistance (HPR) provides an opportunity to manage these issues in GM cotton systems. Cotton cultivars resistant to the sucking bug complex and/or secondary pests would require fewer pesticide applications, reducing costs and risks to beneficial invertebrate populations and pesticide resistance. Incorporation of HPR traits into elite cotton cultivars with high yield and fiber quality offers the potential to further reduce pesticide use and increase the durability of pest management in GM cotton systems. We review the challenges that the identification and use of HPR against invertebrate pests brings to cotton breeding. We explore sources of resistance to the sucking bug complex and secondary pests, the mechanisms that control them and the approaches to incorporate these defense traits to commercial cultivars.

## Cotton – Value As a Crop

Cotton (*Gossypium* sp.) is a major crop in many countries around the world and its fiber is a major raw material for apparel, bed linen, and many other products (Lee and Fang, 2015). About 35 million ha of cotton are planted in the world each year, producing about 26 million tones of lint (ICAC, 2015). The word ‘cotton’ refers to four separate species in the genus *Gossypium* that are grown for the fibers covering the epidermis of their seeds: *G. arboreum, G. barbadense* (Pima cotton), *G. herbaceum*, and *G. hirsutum* (Upland cotton) (Wendel and Cronn, 2001; Wendel and Grover, 2015). This review will focus on *G. hirsutum* cotton, as it comprises around 95% of global cotton production.

## Challenges to Pest Management

Arthropod pests have likely affected cotton since it was domesticated at least 3,000 years ago (Lee and Fang, 2015). A large number of arthropod species have been described as cotton pests, but only less than 40 of them are considered key pests of the crop (Wilson et al., 2013; Luttrell et al., 2015). They directly decrease yield or reduce fiber quality, and their management is a key challenge for cotton growers worldwide. Potential losses up to 40% occur from invertebrate pests alone in cotton (James, 2001; Oerke, 2006). Significantly, even after implementation of control measures, it is estimated that losses of about 12% occur to invertebrate pests (Oerke, 2006). The economic implications of invertebrate pests encompass both crop losses and the costs of control, which mainly consists of insecticides and their application (James, 2001; Naranjo, 2011).

### Domestication and Loss of Plant Resistance to Invertebrate Pests

Plant domestication has successfully increased agricultural productivity supply for humans, although this selection has usually focused on major and highly recognizable traits such as yield and quality, inadvertently losing some others such as adaptation to extreme weather or plant resistance to herbivores (Koricheva, 2002; Macfadyen and Bohan, 2010; Chen et al., 2015). This pattern can be found in the history of the domestication of cotton.

A brief review of the history of domestication in *G. hirsutum* reveals how and why plant resistance traits may have been lost. Although, each of the four domesticated *Gossypium* species has a unique history of domestication and utilization, they were all domesticated in parallel so that the short lint covering the seed was transformed to be a source of textile fiber (Brubaker et al., 1999; Wendel and Cronn, 2001). Following this initial domestication and geographical spread of cotton, some preferred traits were specifically selected, such as: compact and annual growing habits, early maturity, photoperiod neutrality, longer and stronger fiber, and higher yield (more abundant lint on the seed) (Brubaker et al., 1999; Applequist et al., 2001; Gross and Strasburg, 2010). Invertebrate pests probably benefited from selecting cotton plants for increased yield and fiber quality, as this most likely led to trade-offs with the traits controlling invertebrate resistance (Chen et al., 2015). Furthermore, modern high input systems lead to cultivars with higher nutritional value for invertebrates.

The domestication and selection for desirable production and agronomic traits in cotton has gone through phases that have resulted in limited genetic diversity within modern cotton cultivars. Firstly, intense selection during the initial domestication (Iqbal et al., 2001), secondly, industrialization and demand for higher yields of improved-quality cotton meant the US became the focus of cotton germplasm improvement for *G. hirsutum* during the second half of the 19th century (Moore, 1956). Finally, the Mexican boll weevil (*Anthonomus grandis*) appeared in Texas in 1892 causing a significant reduction in cotton production in the southern US. Rapid selection for shorter season cultivars which avoided severe losses to the boll weevil (Smith et al., 1999; Allen, 2008) resulted in a further bottleneck for genetic diversity. There has been some reintroduction of diversity during the last century due importation of genetic stocks of wild *G. hirsutum* cotton imported from Mexico as part of the search for resistance to the cotton boll weevil. However, there are few reports of commercial cultivars with effective plant resistance to sucking bugs, spider mites, aphids, mealybugs or whitefly.

### Reliance on Insecticides and the Genesis of Integrated Pest Management (IPM)

The development and commercialisation of synthetic pesticides (insecticides and acaricides) during the mid-20th century offered highly efficacious and cost effective control of many pests, leading to significant increases in productivity. They also reduced emphasis on selection for traits that may confer resistance to pests. Further, reliance on pesticides lead to selection of pesticide resistance in key pest species, the resurgence of secondary pest outbreaks (e.g., spider mites, aphids) induced by the destruction of natural enemies with pesticides applications (Wilson et al., 1998; Wu and Guo, 2003; Luttrell et al., 2015), elevated costs and environmental contamination (Naranjo, 2011; Wilson et al., 2013). These issues were the catalyst for the development of the IPM approach which considers all available pest control techniques and their combination to reduce both pest populations and reliance on pesticides (FAO, 2015). This can include a wide array of strategies and tactics, e.g., effective sampling, use of economic thresholds, conservation or augmentation of natural enemies and host plant resistance (HPR). Pesticides are an important tool in IPM systems but used primarily to manage pest populations that justify control. The use of pesticides is based on economic thresholds and with preference for use of more selective options that control the target pests but have less negative effect on natural enemies. However, the practical implementation of IPM approaches is often difficult due to the lack of compatibility between conservation of natural enemies and the availability of selective pesticides, as well as to the higher cost of more selective compounds (if available) compared with older broad-spectrum compounds.

### GM Cotton

In many cotton systems the primary pests are lepidopterans such as *Helicoverpa* or *Heliothis* sp., *Earias* sp., and *Pectinophora* sp. Capacity to manage these pests without spraying insecticides would strongly support IPM approaches. Genetic modification (GM) of cotton containing genes to express protein(s) from the bacteria *Bacillus thuringiensis* (*Bt*), which are highly effective at killing the larvae of some lepidopterans (Naranjo, 2011; Wilson et al., 2013), was introduced in the mid-1990s and greatly reduced pesticide use. *Bt*-cotton is highly efficacious against target pests (Lu et al., 2012), at the same time having a negligible effect on non-target insects (Whitehouse et al., 2005, 2014; Tian et al., 2015) and causing little or no harm to most other organisms, including people (Mendelsohn et al., 2003; Herman et al., 2009). Globally, 25 million hectares were planted in 2013 to *Bt*-cotton, representing 68% of all cotton grown in the world. Including other crops, 76 million hectares were planted to genetically engineered crops producing insecticidal proteins from *Bt* (James, 2014).

However, GM cotton is not a ‘perfect’ solution. Firstly, target pest species may become resistant, requiring the implementation of strategies to reduce this risk (Downes and Mahon, 2012). This risk is especially high for cultivars expressing a single *Bt* protein. Several of these genes therefore need to be stacked to delay the development of resistance in the target insect population (Downes and Mahon, 2012; Tabashnik et al., 2013). However, HPR traits may help support resistance management for the *Bt*-cottons as Carrière et al. (2004) and Williams et al. (2011) reported that the presence of the terpenoid gossypol, which provides resistance to a range of cotton pests, can contribute to delaying the development of insect resistance against Cry proteins. Secondly, *Bt*-cotton crops can sometimes provide a more favorable environment for other pests that are not susceptible to the *Bt* proteins. The sucking bug complex in particular was historically controlled co-incidentally by insecticides applied against lepidopteran pests (Naranjo, 2011; Wilson et al., 2013). Consequently, with dramatically reduced pesticide use against lepidopteran pests the sucking pest complex has increased in importance in most *Bt*-cotton systems. These ‘emergent’ pests may require targeted control, which creates further issues as control options are often limited and the less expensive options, such as pyrethroids or organophosphates, are disruptive of natural enemy populations. Use of these compounds against sucking pests ultimately leads to an increase in risks of secondary pests outbreaks, such as spider mites, aphids, or whitefly (Naranjo, 2011; Wilson et al., 2013). These secondary pests then require control, hence, selecting them for pesticide resistance. In Australia for example, spider mites have become resistant to both organophosphates (Herron et al., 1998) and pyrethroids (Herron et al., 2001). Although insecticide applications have greatly decreased with the adoption of *Bt*-cotton, even with the presence of some important outbreaks caused by secondary pests (Naranjo, 2011), some specific situations have been reported with increases in the number of applications required due to these outbreaks (Catarino et al., 2015).

Among the key pests that are challenges in *Bt*-cotton systems are the sucking bugs, spider mites, thrips, silverleaf whitefly, and aphids (Wilson et al., 2013; Luttrell et al., 2015). Sucking bugs are currently considered the primary pest in many of the *Bt*-cotton growing regions such as Australia (Wilson et al., 2013), China (Lu et al., 2010), India (Sharma et al., 2005), and the United States (Naranjo, 2011) and in most seasons will require targeted control. The sucking bug complex comprises primarily of *Adelphocoris* sp., *Lygus* sp., *Creontiades dilutus* and *C. pacificus*, mealybugs (*Phenacoccus solenopsis*, *Pseudococcus corymbatus*, *Pulvinaria maxima*, and *Saissetia nigra*) and the green vegetable bug (*Nezara viridula*). These species feed on young squares and bolls, causing their abortion or damage to developing bolls. Spider mites (predominantly *Tetranychus urticae*) feed on the underside of leaves by sucking out the contents of the mesophyll cells, resulting in reduced yield and fiber quality (Wilson, 1993). Thrips (predominantly *Frankliniella* sp. and *Thrips* sp.) are able to damage cotton seedlings and therefore cause a delay in plant growth and maturity, sometimes reducing yield when the attack is severe (Sadras and Wilson, 1998; Cook et al., 2013). Conversely, later in the season thrips are also considered beneficial insects as they are key predators of spider mites (Trichilo and Leigh, 1986; Wilson et al., 1996; Milne and Walter, 1998). Silverleaf whitefly (*Bemisia tabaci*) secretes honeydew which contaminates lint, causing difficulties in the mill when the fiber is processed (Hequet and Abidi, 2002). The development of silverleaf whitefly populations resistant to a wide range of insecticides exacerbates the problem (Rao et al., 2012). Cotton aphids (*Aphis gossypii*) cause a similar damage to the lint as they excrete honeydew when they feed on the plants. They are vectors for viruses (Ellis et al., 2013) and their feeding distorts plant growth and causes a reduction in photosynthetic activity (Shannag et al., 1998).

## Available Sources and Traits for Host Plant Resistance

Controlling these ‘emergent’ sucking pests with pesticides poses a risk to successful IPM approaches, and at the same time undermines the value of GM technology, as *Bt*-cotton facilitates the control of non-target pests by their natural enemies (Tian et al., 2015). HPR could support sustainable IPM in GM cotton systems by reducing the need to apply insecticides against emergent pests or other secondary pests. Cultivars resistant to key emergent or secondary pests would require less pesticide applications, thus reducing costs, increasing the population of beneficial insects and helping the environment.

### Sources of Resistance in *Gossypium* sp.

The first step to improve HPR to invertebrate pests is to identify the resistance traits that can be incorporated into elite cotton cultivars through breeding. These traits can be found in the cotton genetic pool or created through molecular techniques. Therefore, the availability of gene pools with enough variability to include some genotypes with high levels of HPR is essential. The genus *Gossypium* comprises about 50 species with a high genetic diversity between them. It appeared between 10 and 15 million years ago and diversified in three different centers of origin: Africa–Arabia, Australia, and Central America (Wendel and Grover, 2015). The genus can be divided into eight diploid genome groups (2n = 26 chromosomes), as well as five allotetraploid species (2n = 52). Of these, only four species are grown commercially (*G. arboreum*, *G. barbadense*, *G. herbaceum*, and *G. hirsutum*). The African *G. herbaceum* and the Indian *G. arboreum* are both diploids while the American *G. barbadense* and *G. hirsutum* are both allotetraploids (Wendel and Grover, 2015). The diversity within the cultivated species has declined due to domestication and breeding for increased productivity, as described in Chapter 2. Despite this lack of diversity, especially in *G. hirsutum*, there has been research to identify HPR traits to key pests, summarized in **Table 1**. The bollworm complex has been excluded from the table as this review focuses on management of emergent or secondary pests in *Bt*-cotton systems.

**Table 1:** Genetic sources of host plant resistance and identified traits employed in cotton against pests usually considered as secondary.

Pest	Source of resistance	Resistance trait(s)	Grown commercially (Y/N)	Reference
Sucking bug complex	*Gossypium hirsutum* cultivars and breeding lines	Nectariless plus probably antibiosis	Y	Benedict et al., 1981; Bourland and Myers, 2015
	*G. hirsutum* cultivars and breeding lines	Glandless	N	Leigh et al., 1985
	*G. hirsutum* cultivars and breeding lines	Antibiosis	Y	Tingey et al., 1973
	*G. hirsutum* breeding line	Reduced oviposition preference	N	Tingey et al., 1973
	*G. hirsutum* cultivars and breeding lines	High leaf hair density	Y	Meredith and Schuster, 1979
Spider mites	*G. hirsutum* okra-leaf cultivars	Okra leaf	Y	Wilson, 1994b
	*G. barbadense*	Antibiosis	Y	Schuster et al., 1972; Miyazaki et al., 2012; Zhang et al., 2013
	*G. arboreum* single genotype	Antibiosis	N	Miyazaki et al., 2012
	*G. hirsutum* landraces	Antibiosis	N	Schuster et al., 1972
	*G. australe*	Antibiosis	N	Schuster et al., 1972
	*G. lobatum*	Antibiosis	N	Schuster et al., 1972
Thrips	*G. barbadense*	Unknown, *G.barbadense*-related	N	Zhang et al., 2013
	*G. hirsutum* glandless Acala lines	Glandless	N	Zhang et al., 2014a
	*G. hirsutum* high leaf hair density lines	High leaf hair density	N	Rummel and Quisenberry, 1979
	*G. arboreum* single genotype	Unknown	N	Stanton et al., 1992
	*G. tomentosum*	Tomentum in leaves	N	Zhang et al., 2013
	*G. darwinii*	Not reported	N	Zhang et al., 2013
Silverleaf whitefly	*G. hirsutum* okra leaf genotypes	Reduced feeding preference	N	Chu et al., 2002; Miyazaki et al., 2013a
	*G. hirsutum* glabrous leaf genotypes	Reduced oviposition preference	N	Butler et al., 1991; Miyazaki et al., 2013a
	*G. thurberi*	Okra and glabrous leaves, plus probably antibiosis	N	Walker and Natwick, 2006
	*G. arboreum* single genotype	Antibiosis	N	Miyazaki et al., 2013a, 2014
Jassids or Leafhoppers	*G. armourianum*	Leave thickness, plus probably antixenosis	N	Pushpam and Raveendran, 2006
	*G. raimondii*	High leaf hair density	N	Pushpam and Raveendran, 2006
	*G. hirsutum* selections	High leaf hair density and length	Y	Muttuthamby et al., 1969
	*G. hirsutum* selections	High leaf hair density and length	N	McLoud et al., 2015
	*G. hirsutum* old accessions	Unknown	N	Knutson et al., 2014
	*G. tomentosum*	Tomentum in leaves	N	Knight, 1952

In many of the cases, sources of resistance have been identified but not incorporated to commercial cultivars, probably because of the time and effort that is required. Only in situations where pest control costs have been very extreme or unaffordable (e.g., jassids in India/Africa), has there been a strong effort to breed for HPR (**Table 1**). Sometimes HPR has been identified in the target species, for example high leaf hair density in some *G. hirsutum* populations while in other cases higher HPR have been identified in other cultivated species, for instance *G. arboreum* and *G. barbadense* are more resistant than *G. hirsutum* to some pests such as spider mites and thrips (Miyazaki et al., 2012; Zhang et al., 2014b). Similarly, significant differences have been found in gossypol content between *Gossypium* species (Khan et al., 1999; Stipanovic et al., 2005; Hagenbucher et al., 2013a), and within cotton cultivars (Cai et al., 2010).

Less domesticated populations and wild *Gossypium* species can also be valuable sources of HPR traits. Resistance to various cotton pests have been reported in these diploid cottons (**Table 1**), though in many cases the cause of resistance is unknown. These include; *G. arboreum* against thrips and spider mites (Stanton et al., 1992; Miyazaki et al., 2012), *G. armourianum* and *G. raimondii* against jassids (Pushpam and Raveendran, 2006), *G. australe* and *G. lobatum* against spider mites (Schuster et al., 1972), *G. darwinii* against thrips (Zhang et al., 2013), *G. tomentosum* against jassids and thrips (Knight, 1952; Zhang et al., 2013), *G. thurberi* against whitefly (Walker and Natwick, 2006) and *G. trilobum* against spider mites and silverleaf whitefly (Miyazaki et al., 2012, 2013a). However, introgression of resistance from wild species is a very long process and sometimes unsuccessful due to the difficulty of introducing HPR traits from a diploid into a tetraploid (Ganesh Ram et al., 2008), usually by creating a synthetic tetraploid, while improving or maintaining yield and fiber quality. Landraces and old cultivars may also offer valuable HPR traits, and as they are tetraploid the process of introgression is significantly shorter. The value of all of these underutilized *Gossypium* genetic resources will be reinforced with the development of new molecular techniques which will greatly enhance the introgression of the resistant traits into commercial cultivars.

### Plant Defense Mechanisms

Host plant resistance against herbivorous invertebrate pests is generally defined as “the sum of genetically inherited qualities that results in a plant of one cultivar or species being less damaged by a pest arthropod than a susceptible plant lacking these qualities” (Panda and Khush, 1995; Smith, 2005). Among its benefits as a pest control measure, HPR is durable, easy to use, environmentally friendly and compatible with other management practices (Smith, 2005; Wilson et al., 2013). On the other hand, breeding for HPR is generally a slow and difficult process that has mostly been overlooked in preference to use of chemical control of pests. In recent times, breeding for HPR is becoming a more feasible alternative due to several facts: the reduction in the impact of the Lepidopteran pests by *Bt-*cotton, increasing pest resistance to insecticides, enactment of strict environmental regulations on insecticides and their use, and advances in molecular technologies.

Plant defense mechanisms have been traditionally classified into three main categories (Painter, 1958; Panda and Khush, 1995; Smith and Clement, 2012): antixenosis or non-preference mechanisms, that prevent or deter the herbivore from feeding on the plant; antibiosis mechanisms, that affect the insects performance and survival by a physical or chemical trait; and tolerance, that represents the plant’s ability to compensate for herbivore damage and yield productivity. Currently, tolerance is usually regarded as a plant defense strategy separate from resistance (Rosenthal and Kotanen, 1994; Núñez-Farfán et al., 2007). Resistance is to cover “those plant traits that reduce the extent of injury done to a plant by a herbivore” as in practice antixenosis and antibiosis are often difficult to separate (Stout, 2013). Resistance mechanisms or categories can also be direct (e.g., antibiosis, leaf morphology) and indirect (e.g., attraction of natural enemies of the herbivore), and they can be expressed constitutively (e.g., leaf morphology) or be induced following a cascade of processes after some damage is caused by the herbivory (e.g., induced chemical responses) (Schuman and Baldwin, 2016). All of these mechanisms are unusually controlled polygenetically (Stout and Davis, 2009; Smith and Clement, 2012), but a number of cases of single-gene resistance have also been reported (Kaloshian, 2004; Stuart, 2015).

### HPR Traits Available in Cotton

Traits providing HPR in cotton can include one or several defense mechanisms functioning in a complex way. Some of the morphological traits provide a mechanical barrier to the pest, such as trichomes or hairs on leaves, while others influence the general growing habit and appearance of the plant, such as okra leaf or red coloration of the plant (Jenkins and Wilson, 1996; Wilson and Sadras, 1998) or even the microclimate conditions present on the leaf, such as in okra leaves (Wilson, 1994b). There is also a wide array of chemical compounds used by cotton plants to defend themselves from herbivores, such as flavonoids, tannins and particularly terpenoids such as gossypol (Wink, 1988; Sadras and Felton, 2010; Hagenbucher et al., 2013a). The latter is produced by plants of the genus *Gossypium* and has been shown to be toxic to many pests that affect cotton (Jenkins and Wilson, 1996; Cai et al., 2010; Hagenbucher et al., 2013a). The application of HPR traits is complex as different traits can operate at the same time to provide a given level of resistance. A number of reviews focused on HPR traits in cotton are available (Jenkins and Wilson, 1996; Wilson and Sadras, 1998; Sadras and Felton, 2010; Hagenbucher et al., 2013a). In the present review, HPR traits will be discussed from the point of view of the genetic source providing the resistance and the prospects for the incorporation of these traits in commercial cultivars.

Traits for direct resistance mechanisms are frequently targeted in HPR breeding because they usually have major effects and they are also easier to identify and select for. On the other hand, traits for indirect HPR are not as simple to identify and are rarely targeted. Traits for both constitutive and induced HPR can play a major role controlling HPR, but constitutive mechanisms are more usually targeted as once they are identified, plants carrying them can be selected without having to perform a bioassay. For that reason, traits for constitutive morphological resistance, such as a high leaf hair density or thickness are often initially targeted in breeding programs. Other traits for constitutive HPR, such as constitutive chemical compounds, can also be relatively simple to target. However, the initial identification of the specific compounds involved in the resistance is often more challenging than identifying morphological HPR traits. Antibiosis traits can have the biggest impact on HPR and are probably the most successfully used in cotton, both in breeding for secondary pests (**Table 1**) and in main pests (*Bt*-cotton). However, identifying antibiosis is not as straightforward as other HPR traits such as morphological traits, often requiring the use of bioassays.

### Using HPR Traits against Emergent and Secondary Pests in Cotton

Although, not an emergent pest in *Bt*-cotton systems, the cotton boll weevil has historically been the catalyst for considerable effort toward selection of HPR genotypes (Bourland and Myers, 2015). In areas where it was a pest there was a shift in the cultivated germplasm toward short-season early maturing cultivars to reduce the period of exposure to the pest (Smith et al., 1999). Cotton boll weevil has since been eradicated from most areas of the eastern USA and this has allowed a significant increase in cotton productivity in these areas (Allen, 2008). Unfortunately, cotton boll weevil is causing major challenges to cotton production in some parts of South America, especially in Brazil where it is currently considered the most important cotton pest (Lima et al., 2012).

Resistance to spider mites has been studied and reviewed by Wilson and Sadras (1998) and Miyazaki et al. (2012, 2013b). Okra leaf (Wilson, 1994b) has been related to an increased resistance to this pest. However, biochemical traits seem to offer more effective resistance, as reported for *G. arboreum* and *G. barbadense* genotypes (Miyazaki et al., 2013b) and some *G. hirsutum* landraces (Schuster et al., 1972; **Table 1**).

*Gossypium barbadense* cultivars possess a major gene conferring a higher level of resistance to thrips, according to the segregation of resistant plants reported by Zhang et al. (2013). Glandless cotton (no gossypol glands; Zhang et al., 2014a) and high leaf hair density genotypes (Rummel and Quisenberry, 1979) have also been reported to provide some level of HPR to thrips, but the exact mechanisms have not been studied. Tolerance or compensatory responses have also been reported in damaged cotton seedlings by thrips (Sadras and Wilson, 1998; Wilson et al., 2003).

Several morphological traits have been associated with partial resistance to silverleaf whitefly. Okra shaped leaves (Chu et al., 2002), and very smooth (glabrous) or very hairy leaves harbor less whiteflies than moderately hairy leaves (Butler et al., 1991; Miyazaki et al., 2013a). Very high level of resistance against SLW has been reported in the wild diploid species *G. thurberi* (Walker and Natwick, 2006), which has both okra and glabrous leaf traits. Whitefly resistance has also been associated with biochemical traits, and particularly with the amount of total sugars, tannins, flavonoids, phenols, and gossypol (Butter et al., 1990).

Regarding the sucking bug complex, compensatory or tolerant responses have also been reported in later stages of the plant for damage caused by *Lygus* sp. (Barman and Parajulee, 2013) and *Creontiades dilutus* (Duggan et al., 2007), although the effect of the genotype was not studied. Nectariless (absence of glands exuding nectar) cotton genotypes have been reported to harbor lower plant bug populations (Benedict et al., 1981; Bourland and Myers, 2015). High leaf hair densities have also been reported to provide a higher level of resistance (Meredith and Schuster, 1979). High leaf hair density has also been associated with resistance to the cotton jassid or leafhoppers (Muttuthamby et al., 1969; Bhat et al., 1982; McLoud et al., 2015), as it interferes with oviposition.

With the exception of the nectariless trait, indirect mechanisms of HPR have never been targeted in cotton, and rarely in other crops (Wäckers, 2005). However, there are some new promising achievements in this field, such as the selection of maize plants with a high emission of induced plant volatiles that attract natural enemies of the target pest (Tamiru et al., 2015). Further exploration of these mechanisms in cotton genotypes may be worthwhile within an IPM strategy.

## Breeding Approaches for Resistance to Emerging and Secondary Pests

There is sufficient genetic diversity to warrant HPR breeding programs to a range of emerging pests within *G. hirsutum* and its primary and secondary gene pools. The success of HPR breeding, as for any other program, depends on the complexity of the inheritance of the trait and the ease and reproducibility of the phenotype. The major additional complication for breeding for HPR is that it is essential to understand the nature of the resistance, and the potential benefits and risks from that characteristic. Resistance mechanisms often mean a trade-off for the plant, either among these mechanisms and other plant traits (Strauss et al., 2002), or among different defense mechanisms working on the plant (Kariñho-Betancourt and Núñez-Farfán, 2015), which has also been demonstrated in cotton (Rudgers et al., 2004). For instance, resistance to one pest may result in increased susceptibility to other pests, such a leaf hairness which provides resistance against jassids (Muttuthamby et al., 1969) but can make plants more susceptible to spider mites (Wilson and Sadras, 1998). Ecological interactions are also important as HPR traits can reduce a target pest but also negatively affect beneficial populations, such as the nectariless trait where leaves do not develop the extraflora nectaries, making the cotton less attractive to plant bugs but also reducing abundance of beneficials species that use nectaries as supplementary food (Adjei-Maafo and Wilson, 1983). This result suggests that some HPR traits can lead to ‘enemy-free space’ and thereby inadvertently advantage a non-target herbivore species (Hagenbucher et al., 2013b). Interactions at multitrophic levels must also be considered as HPR traits may directly affect both beneficials and non-target herbivores. For instance, the presence of extrafloral nectaries can attract and increase the population of natural enemies by providing them food (Adjei-Maafo and Wilson, 1983; Wäckers, 2005) but can also enhance the fitness of some herbivores, such as plant bugs, or make the crop more attractive for oviposition of *Helicoverpa punctigera* moths that also use nectar as a supplementary food source (Benedict et al., 1981; Flint et al., 1992). Nevertheless, most commercial *G. hirsutum* varieties have extrafloral nectaries.

Interactions between HPR traits, GM traits and herbivores are also important. In most *Bt*-cotton systems the sucking bug complex has become more important, requiring targeted control with insecticides. The cause of this increased pest status may be partially due to ‘insecticide release’ as they are no longer being coincidentally controlled by insecticide applications targeting lepidopteran pests (Naranjo et al., 2008). However, it has also been suggested that competitive release of the plant bug complex from competition with lepidopteran pests is also a possible contributing factor to increases in abundance of sucking bugs in *Bt*-cotton systems (e.g., Whitehouse et al., 2007; Zeilinger et al., 2011) or because *Bt*-cotton plants have less induced production of terpenoids due to reduced feeding damage from lepidopteran larvae (Hagenbucher et al., 2013b). In any case this example highlights the potential complexity and hence capacity for unexpected changes that could occur when combining GM and HPR traits.

Some traits come at a high metabolic cost or altered phenology that lowers yield, such as use of short season cultivars to avoid pest attack, or result in an unwanted side effect, for instance- high leaf hairiness is incompatible with mechanized picking (Anthony and Rayburn, 1989), and gossypol in the seed is toxic to animals that are fed with cottonseed (Berardi and Goldblatt, 1980). However, the presence of gossypol has been removed by breeding glandless cotton cultivars (Cai et al., 2010), though these are more susceptible to invertebrate (both the fruit and leaves; Jenkins et al., 1966) and vertebrate pests (mice attacking seeds). A more effective approach has been the development of ultra-low gossypol cottonseed GM varieties, where gossypol production is selectively inhibited in the seeds but not in the rest of the plant (Rathore et al., 2012). Due to these issues, breeding for HPR is usually regarded very cautiously and a cost/benefit analysis must be applied to determine what HPR traits are targets for introgression into elite cultivars.

### Identifying New Sources of HPR

Identifying new sources of resistance by phenotyping involves exposing a range of cotton genotypes to the pest population, either in the field, greenhouse or laboratory and assessing some measure of pest fitness (developmental rate, survival, fecundity, life span) and/or plant damage – essentially a large scale bioassay. Selection of genotypes can be directed by previous published literature, however, these studies are limited and mechanisms involved in the HPR reaction are not always reported. If there is no useful resistance available amongst domesticated *G. hirsutum* genotypes, the range of material tested will need to be expanded to include race lines and other *Gossypium* species. Once material has been assembled, experiments need to be set up in the field or greenhouse to evaluate pest fitness and plant damage responses. This can be challenging as the pest may not reliably appear at densities sufficient to discriminate between cotton genotypes, and experiments may require significant amounts of land or greenhouse space to allow a realistic number of genotypes to be evaluated with sufficient replication for the results to be statistically reliable. Non-target pest species may invade the experiments and require selective management and beneficial species may reduce pest abundance.

Culturing pests and releasing them onto candidate genotypes, either in the field, greenhouse or laboratory is an approach that has been used to ensure sufficient pest density with some success (Wilson, 1994a; Parajulee et al., 2006). This ensures more reliable results, but cultures must be maintained, keeping them free of other pest contaminants (e.g., spider mites in aphid cultures), free of problems with beneficial invertebrates attacking the pests (e.g., aphid or whitefly parasitoids invading cultures or field experiments) and vigorous so that they accurately represent the likely behaviors of ‘wild’ populations. Research in greenhouse situations can be indicative of field performance but conditions may mask differences in microclimate (Wilson, 1994b) and plants may perform differently in the field and greenhouse, such as differences in expression of leaf hairiness between field and greenhouse grown plants (Miyazaki et al., 2013a).

In an ideal situation the performance of the candidate genotypes would be evaluated under protected (no pests) and unprotected (pests present) scenarios to assess the resistance of the genotypes to the pest by comparing pest abundance and relative yield between protected and unprotected treatments. This again creates challenges with logistics of sampling pest abundance, managing other pests, land, labor and costs. These issues are all manageable in the search for sources of resistance, however, once resistance has been identified and a breeding program initiated to introgress traits into more desirable genetic backgrounds there is a need to screen many genotypes at successive stages in the HPR trait introgression process. In this situation the screening of genotypes in bioassays to confirm resistance to pests can quickly become a limiting factor.

Plant phenotyping for HPR is therefore a key limiting factor and improving the speed and accuracy is crucial to develop genotypes with effective HPR. High-throughput phenotyping using automation, robotics and remote data collection is changing the way cultivars are developed (Goggin et al., 2015). These new techniques can speed up the process of collecting and analyzing data, but the use of bioassays, with all their issues identified above, is still necessary. Eliminating a large proportion of genotypes early in the breeding process without the need of bioassays is therefore still desirable and might be possible by genotyping. New molecular tools could help in fulfilling this need, thus speeding up the HPR conventional breeding process, however, the HPR traits still need to be identified and characterized prior to the use of molecular tools.

### Molecular Tools to Complement Phenotyping of HPR Traits

Once potential HPR traits have been identified, modern molecular techniques, which are evolving at a rapid pace, provide the opportunity to dramatically expedite breeding by avoiding the need to constantly assess the presence of HPR traits in genotypes by bioassay. The difficulty of bio-assaying for some HPR traits makes the identification of molecular markers that are closely linked to HPR traits and can be used a substitutes for performing HPR bio-assays, essential for breeding. The completion of the draft genome sequence for *G. hirsutum* cultivar TM-1 (Li et al., 2015; Zhang T. Z. et al., 2015) marks a major milestone as it facilitates a number of molecular assisted breeding strategies that can speed the identification of molecular markers linked to HPR traits. Next generation sequencing technologies and high throughput genotyping technologies has expedited the creation of high density genetic maps in cotton that have resulted in the identification of the causal gene for okra leaf (Zhu et al., 2015). The genes for other genetically simple HPR related traits such as nectariless and frego bract will be soon identified, resulting in “perfect” molecular markers that can used as a diagnostic for the traits in young plants or seeds. In other species, several genes have been already identified as conferring HPR, for instance HPR in rice to brown planthopper (*Nilaparvata lugens*) provided by genes *Bph14* (Du et al., 2009) and *Bph3* (Hogenhout and Zipfel, 2015).

As the desired HPR is often found in agronomically poor germplasm, additional molecular markers located either side of the causal gene allows breeders to select for plants that contain little or no linkage drag that has often masked the benefits of an introgressed trait. Large scale genotyping platforms such as the Illumina CottonSNP63K array can readily identify chromosomal segment substitutions. Therefore by repeated backcrossing of the trait into an elite cultivar, linked markers to the trait(s) can be found after only a few rounds of backcrossing. Confirmation that the donor regions are linked to resistance can be performed in a further cycle of backcrossing, selfing and selection for resistant lines. This strategy is especially useful when traits are obtained from the secondary gene pool via synthetic tetraploid bridges. High throughput genotyping also makes possible obtaining linked markers via genome wide association studies on a range of cultivars and their pedigrees containing different levels of HPR, which avoids the time and energy required in the creation of specialized genetic populations. However, a robust and reliable phenotyping will still be necessary as the level of resistance needs to be confirmed in bioassays with the target pest during the discovery and validation phases.

### Challenges and Potential Opportunities with Complex Traits

Marker assisted selection has generally been found to work well for simple genetic traits, or regions that exert a major quantitative influence, but have proven ineffective for genetically complex traits comprising many loci of small effect (Desta and Ortiz, 2014). Although, few quantitative genetic HPR analyses have been performed in cotton, from other plant systems it is thought that many important HPR traits are genetically complex (Stout and Davis, 2009; Smith and Clement, 2012). Genomic selection, a form of marker-assisted selection (Heffner et al., 2009) that has only recently became feasible in cotton, can enable genetically complicated HPR traits to be incorporated into elite cultivars (Desta and Ortiz, 2014). Genomic selection requires large populations to be accurately phenotyped and genotyped, such that there are markers covering the whole genome so that all genes are in linkage with at least one marker. The aim of genomic selection is to computationally predict genomic estimated breeding values, first by analyzing a training population composed of plant lines covering all important germplasm (i.e., founders) in the breeding program, and then validating the models on subsequent breeding populations. The advantage of this methodology is that it takes into account many regions which have a small effect from the different backgrounds of the breeding populations targeted. Genomic selection therefore has the ability to optimize the HPR of cultivars using existing variation within the breeding population.

### New Methodologies for Generating and for Introgressing HPR Traits

There is significant scope for improving HPR by marker assisted breeding but introgressing traits from distant germplasm such as from the secondary gene pool, still remains a challenge and requires generations of crossing and selection. It also precludes acquiring HPR from the tertiary gene pool that consists of diploid *Gossypium* species with a completely different genome type that generally show poor or no recombination with *G. hirsutum*. To access HPR traits from these species will require identification of the causal gene. These genes can then be transferred into cultivated *G. hirsutum* cotton by GM or gene editing technology. GM traits are subject to complex and expensive regulatory systems, that cannot be grown in some countries (Tabashnik et al., 2013; James, 2014) and so the HPR trait must possess a significant economic value to compensate for the regulatory investment. The regulatory status of genome editing is currently unknown, but as simple genome edits are indistinguishable from natural or induced mutations there is the possibility that that these plants may not be subject to the same strict regulations as GM cotton. Genome editing might prove be the main avenue for acquiring HPR from diverse *Gossypium* species, especially as both the A_t_ and D_t_ genomes present in *G. hirsutum* should be able to be edited simultaneously (Wang et al., 2014).

Natural genetic diversity for HPR against a pest is not always available or easily accessible. In such cases, new diversity can be induced using chemical mutagens, ionizing radiation or transposable elements. Mutation breeding of *G. hirsutum* has resulted in ‘naked and tufted’ seeds, herbicide resistance and plants with longer fiber (Auld et al., 2007; Bechere et al., 2009a,b) and may provide a means of obtaining novel forms of HPR especially via developmental or secondary metabolism changes.

The history of breeding for HPR against Lepidopteran pests illustrates that for some pests adequate control can only be achieved by using GM technology to access resistance that have evolved in other biological systems. There are a number of promising GM avenues that may help control the rise of emergent and secondary pests in *Bt*-cotton. Sap-sucking insects (Hemipterans) are generally not susceptible to *Bt*, however, Chougule et al. (2013) added a short pea aphid (*Acyrthosiphon pisum*) gut binding peptide to Cry2Aa that resulted in enhanced toxicity to both pea aphid and green peach aphid. A thorough understanding of the binding and mode of action of the Cry toxins may enable modified toxins to specifically target other important pests. Secondary plant metabolites are also a source of potential resistance (Birkett and Pickett, 2014). Small lipophilic molecules are a promising group of secondary metabolites that can have similar physiochemical properties and toxicities to pesticides or insect pheromones. These metabolites pathways can be engineered into plants to help manage pests, although the metabolic pathways are complex and may be energy intensive leading to a trade-offs with yield (Birkett and Pickett, 2014).

The discovery that ingested double stranded RNA can trigger RNA interference (RNAi) in nematodes (*Caenorhabditis elegans*) has opened up the possibility of plants expressing targeted RNA species that could silence essential genes in pest species resulting in their death or reduced fecundity (Fire et al., 1998). Mao et al. (2011) found that cotton plants expressing a dsRNA that targets a *Helicoverpa armigera* P450 monooxygenase gene (*CYP6AE14*) associated with detoxification of gossypol, resulted in reduced growth of bollworms and less plant damage. Yue et al. (2016) found that cotton expressing dsRNA against a *H. armigera* gene involved in feeding behavior, resulted in significantly reduced leaf damage and smaller larval body size. This technology has the potential to be selective as it is based on the sequence of its target sequence, thus no effect should be observed on non-target species. The difficulties associated with the technology involve the selection of target genes that are required for a vital process to the pest species, and delivering the dsRNA at levels that are effective (Miller et al., 2012) as these RNAi plants usually inhibit, but do not kill, their target host (Mao et al., 2011; Zha et al., 2011). Expression of dsRNA in chloroplasts has resulted in higher levels of these transcripts and better efficacy against target insects (Jin et al., 2015; Zhang J. et al., 2015). However, plastid transformation is only possible in a limited number of plant species and is not currently practical in cotton. Foliar application of dsRNA targeted to pest species is also currently being explored as a novel form of insecticide. It is possible that this method of delivery will become more prevalent than GM, as it avoids plant registration costs, is more flexible and appears relatively stable (San Miguel and Scott, 2015).

## Conclusion

The history of cotton production is linked with the history of the emergence of new pests. In recent times, these emergence events have generally been related to the use of insecticides and/or the emergence of *Bt*-cottons (Luttrell et al., 2015). However, there are few examples of successful deployment of HPR traits to the emergent pests or linked secondary pests in cotton cultivars. Recent research indicates that there is significant scope to improve HPR in cotton especially against key secondary pests. This review outlines sources of germplasm and the opportunities to improve HPR in cotton against invertebrate pests in GM cotton systems. Unfortunately, traits providing a high level of HPR sometimes have other undesirable effects. Therefore, it is necessary to use caution when introgressing these HPR traits into elite cultivars. Modern techniques can also help to identify and expedite the process of incorporating HPR traits into elite germplasm.

Some caution is also required, as there is a risk that the target population of herbivores can overcome the improved defense mechanisms of the plant, leading to an “arms race.” Lessons from the development of pesticide resistance in many insect and mite species suggest that any HPR mechanism which is based on a single toxin affecting pest fitness would impose strong selection for resistance in the target pest population. Issues with emerging resistance in *Bt*-cottons reinforce this fact and highlight the need of integration of HPR within IPM tactics.

Ultimately, the success of incorporating HPR will depend on the benefit it can provide compared with current strategies to manage the pest and any potential agronomic cost in terms of yield and fiber quality compared with elite cultivars. Nevertheless, HPR represents an opportunity to improve the value to cotton production systems that the current pest resistant *Bt*-cottons offer.

## Author Contributions

CT, WS, and LW conceived and designed the review. CT drafted the review. CT, IW, WS, and LW wrote the review.

## Conflict of Interest Statement

The authors declare that the research was conducted in the absence of any commercial or financial relationships that could be construed as a potential conflict of interest.
